# Age and Race-Related Differences in Sleep Discontinuity Linked to Associative Memory Performance and Its Neural Underpinnings

**DOI:** 10.3389/fnhum.2019.00176

**Published:** 2019-06-04

**Authors:** Emily Hokett, Audrey Duarte

**Affiliations:** Memory and Aging Lab, Department of Psychology, Georgia Institute of Technology, Atlanta, GA, United States

**Keywords:** sleep quality, episodic memory, neural oscillations, alpha, aging, diversity

## Abstract

There is a strong relationship between sleep and memory for the details of past events. In old age, both episodic memory performance and related neural activity decline. These changes occur in parallel to age-related decreases in sleep quality. Thus, poor sleep quality may be an explanatory factor for poor memory in older adulthood. Furthermore, Black adults tend to sleep more poorly than White adults, and this could be explained by differences in health and psychosocial factors (e.g., socioeconomic status, race-related stress). However, there have been no studies investigating the effect of race on sleep quality, episodic memory, and memory-related neural function. In the current pilot study, we recruited a diverse sample of older and younger adults and measured their habitual sleep using a wrist-worn accelerometer for 1 week. We recorded their electroencephalography (EEG) as they performed an episodic memory task to assess the impact of habitual sleep on memory-related neural oscillations. We found that more variable sleep quality was associated with worse memory performance, particularly for older adults. Additionally, Black participants demonstrated greater intraindividual sleep variance than White participants, and greater sleep variance was strongly linked to reduced memory-related neural activity in Black participants. Taken together, maintaining good sleep quality is especially important for memory performance in older adulthood, and greater sleep variation, that is evident in Black adults, may hamper memory-related neural function.

## Introduction

The importance of sleep for memory consolidation has been firmly established (for a review, see Rasch and Born, 2013). Sleep manipulation studies (sleep vs. wake, sleep deprivation) show that episodic memory, or memory for previously experienced events, is sleep dependent in young and older adults (Yoo et al., 2007; Aly and Moscovitch, 2010; Payne et al., 2012; Wilson et al., 2012). Lab-based polysomnography studies have identified electroencephalography (EEG) sleep signatures that are indicative of memory consolidation (for a review see, Mander et al., 2017). It has been found that sleep spindles predict subsequent memory-related hippocampal activity in young and older adults (Mander et al., 2013). However, such studies do not allow for assessments of natural sleep patterns, from the comfort of one’s own home, nor do they typically monitor sleep over multiple nights. This type of measurement is necessary to examine habitual sleep quality, which has been tied to poorer memory in older adults (Wilckens et al., 2014; Cavuoto et al., 2016).

Meta-analyses and large studies have shown that older adults, as compared to young adults, are more likely to experience chronic sleep disruptions that include reduced total sleep time (TST) and increased wake after sleep onset (WASO; Carrier et al., 2001; Ohayon et al., 2004). There is also evidence that older adults experience more night-time awakenings (Dijk et al., 2001). Prior research suggests that older adults are not as affected by sleep as young adults are (Gui et al., 2017); however, these studies are typically limited to sleep-wake comparisons. Such broad manipulations do not capture individual differences in habitual sleep quality that may be more sensitive to memory in older adults. Importantly, individual differences in sleep quality measures, that can be captured by actigraphy, have been linked to individual differences in episodic memory performance, particularly in older adults (Mary et al., 2013; Wilckens et al., 2014; Seelye et al., 2015; Cavuoto et al., 2016). For example, research has shown that habitual sleep discontinuity, calculated as mean sleep disruption over time, (e.g., WASO) disproportionately affects memory recall in older adults (Wilckens et al., 2014). Subjective measures of sleep discontinuity have illustrated similar relationships; the self-reported number of night time awakenings has been negatively linked to associative memory recall in older, but not young adults (Mary et al., 2013). This limited evidence suggests that older adults are particularly sensitive to mean sleep discontinuity, but the relationship between night-to-night intraindividual variance in sleep discontinuity has not been studied.

While age-related differences in the effect of habitual sleep quality on episodic memory has been investigated, race-related differences in these effects are largely unexplored. The majority of research investigating age-related changes in sleep and memory has either not reported racial demographics of study participants or has been limited to predominantly White participant samples. Importantly, however, population studies have consistently reported poorer sleep in minorities, including Black and Latino Americans, than White individuals (Hicken et al., 2013; Slopen and Williams, 2014; Cunningham et al., 2016; Turner et al., 2016). Education has been related to sleep quantity and quality in minorities (Hicken et al., 2013; Slopen and Williams, 2014; Turner et al., 2016), but this relationship is thought to be confounded by variables associated with lower education such as poorer living and working conditions (Bixler, 2009). Moreover, a few studies have shown that after adjusting for education, psychosocial factors, particularly discrimination and race-related stress, explain racial/ethnic differences in sleep quality (Hicken et al., 2013; Slopen and Williams, 2014). However, it is important to note that these studies subjectively measured sleep quality with short questionnaires. Thus, it remains unknown if similar racial differences exist for objectively measured sleep quality and the extent to which such differences contribute to individual differences in memory performance.

The underlying neural oscillations involved in episodic memory have not been explored in relation to habitual sleep quality. Neural oscillations in the alpha and theta frequency have been consistently linked to memory performance (for a review see, Hanslmayr et al., 2012; Hanslmayr et al., 2016). One such study found that alpha desynchronization increases as a function of the amount of successfully retrieved information (Khader and Rösler, 2011). Furthermore, compared to restful sleep, acute sleep restriction has been shown to reduce retrieval-related alpha desynchronization (Alberca-Reina et al., 2015). Thus, the research demonstrates that sleep is related to waking, memory-related neural activity. However, there is an absence of research examining these effects in relation to habitual sleep quality, or sleep over time, which emphasizes the necessity of the present pilot study.

The current study is the first to examine interactions between age and race on both the mean and intraindividual variance of habitual sleep quality and their contribution to episodic memory performance and related neural activity. Given evidence showing that sleep affects memory performance and memory-related neural activity during episodic retrieval (for a review see, Kreutzmann et al., 2015), we hypothesize that poorer and more variable habitual sleep quality will predict worse memory performance and reduced memory-related neural oscillations. Furthermore, considering the well-established reductions in sleep quality in older adults and minority groups, poor sleep quality may disproportionately relate to memory performance in older, Black participants.

## Materials and Methods

### Participants

Eighty-one participants (45 older, 36 younger) from the Georgia Institute of Technology and the Atlanta community enrolled for the present study. Young adults were recruited within the age range of 18 to 37 years, and older adults were recruited from 56 to 76 years. All participants were right-handed, native English speakers, with normal or corrected to normal vision, free of uncontrolled psychiatric disorders, neurological disorders, sleep disorders, and vascular disease. All participants signed consent forms approved by the Georgia Institute of Technology Institutional Review Board.

Sleep data for all 81 participants were used for a principal components analysis (PCA) of sleep data (see section “Principal Components Analysis”). Eleven of the 81 subjects were excluded from further analyses because they were run on an early procedure in which a delay was placed between encoding and retrieval, 2 did not finish the experiment, 7 had insufficient numbers of trials for EEG analysis (i.e., fewer than 10 misses or high confidence hits), and 11 did not identify as either Black or White. The age and racial breakdown of the final sample are shown in Table 1.

**Table 1:** Group characteristics by age and race.

Measure	Young		Old				
					
	Black (*n* = 11)	White *(n* = 9)	Black (*n* = 9)	White (*n* = 21)	1	2	3
Age	25.09 (1.71)	22.89 (1.36)	63.33 (1.65)	69.42 (0.97)	^∗∗^	^∗∗∗^	
Gender (Women/Men)	6/5	6/3	4/5	12/9			
Education	15.00 (0.38)	15.44 (2.79)	15.22 (0.70)	15.29 (0.46)			
High Confidence *d’*	1.69 (0.20)	1.65 (0.77)	1.33 (0.23)	1.04 (0.17)		^∗^	
High Confidence Trials	0.67 (0.05)	0.64 (0.03)	0.73 (0.05)	0.75 (0.04)			
List Recognition	11.75 (0.16)	12.00 (0.00)	11.50 (0.27)	12.00 (0.00)			^∗^
Visual Recognition	16.82 (1.27)	17.67 (1.27)	13.11 (1.86)	15.43 (0.92)		^∗^	
Verbal Span	12.64 (0.62)	11.33 (1.00)	9.22 (0.74)	10.95 (0.55)		^∗^	
Trials A (seconds)	25.62 (2.51)	22.85 (1.92)	35.19 (3.00)	35.63 (2.71)		^∗^	
Trials B (seconds)	66.91 (9.77)	54.45 (5.86)	101.50 (13.01)	67.67 (2.85)		^∗^	^∗^
MOCA	27.55 (0.37)	28.00 (0.55)	23.33 (1.01)	27.40 (0.41)	^∗∗∗^	^∗∗∗^	^∗∗^
DASS-Stress	3.00 (1.12)	4.78 (1.28)	2.78 (0.98)	3.52 (0.71)			
IRRS-B Cultural	28.75 (5.12)	21.25 (3.12)	28.00 (7.08)	14.33 (2.20)			^∗∗^
IRRS-B Institutional	10.50 (2.40)	9.75 (2.17)	12.25 (3.54)	6.50 (0.50)			^∗^
IRRS-B Individual	11.25 (3.35)	7.50 (1.50)	13.33 (2.40)	6.44 (0.44)			^∗^
Income			33434.67 (4851.62)	59856.71 (4013.46)	^∗∗^		
TST-M	397.93 (20.32)	417.89 (17.92)	350.95 (15.04)	411.37 (11.39)			^∗^
TST-V	12706.23 (4561.47)	5047.56 (1200.78)	5046.26 (1552.16)	3889.18 (978.34)			^∗^
Number of Awakenings-M	15.70 (2.15)	16.16 (2.42)	17.96 (2.71)	13.23 (0.97)			
Number of Awakenings-V	43.31 (9.00)	23.70 (4.40)	25.28 (6.22)	20.20 (4.20)			
Sleep Efficiency-M	87.27 (1.51)	88.65 (2.84)	82.55 (3.90)	89.64 (0.90)			^∗^
Sleep Efficiency-V	29.94 (8.41)	25.71 (16.22)	34.22 (15.88)	21.01 (4.24)			
WASO-M	49.14 (6.43)	47.53 (13.84)	74.17 (19.49)	43.80 (4.48)			
WASO-V	841.74 (290.19)	557.76 (367.69)	596.88 (139.94)	660.88 (181.70)			
SFI-M	29.48 (3.01)	24.03 (4.03)	40.26 (5.16)	24.67 (1.25)			^∗∗^
SFI-V	134.31 (31.57)	90.43 (18.42)	169.09 (33.03)	114.24 (18.28)			
Sleep Discontinuity Component - M	0.06 (0.28)	-0.03 (0.34)	0.57 (0.51)	-0.26 (0.13)			
Sleep Time Component- M	0.05 (0.31)	0.47 (0.34)	-0.63 (0.22)	0.23 (0.17)		^∗^	
Sleep Discontinuity Component-V	0.39 (0.35)	-0.20 (0.25)	-0.32 (0.22)	-0.30 (0.22)			
Sleep Time Component - V	0.32 (0.46)	-0.33 (0.21)	0.35 (0.43)	-0.14 (0.17)			


*The standard error of the mean is in parentheses. Mean differences between old Black and old White are in column 1; age group main effects are marked in column 2; and racial group main effects are in column 3. There were no significant differences between young Black and young White participants. The IRRS-B measures are based on a smaller subset of participants. TST = Total Sleep Time; WASO = Wake After Sleep Onset; SFI = sleep fragmentation index; M = mean; V = variance. ^∗^*p* < 0.05, ^∗∗^*p* < 0.01, ^∗∗∗^*p* < 0.001.*

### Procedure

Participants were administered a battery of standardized neuropsychological tests to rule out mild cognitive impairments. The test battery consisted of subtests from the Memory Assessment Scale (Williams, 1991) including list recognition, visual recognition, and verbal span. Participants also completed Trials A and B, a subtest of the Halstead-Reitan Neuropsychological Test Battery (Reitan and Wolfson, 1985). Lastly, the Montreal Cognitive Assessment (MOCA; Nasreddine et al., 2005) was administered to further test for mild cognitive impairments. The MOCA cutoff score of 26 was used as a guide in conjunction with the other neuropsychological tests, as the MOCA was developed in Montreal and may not be able to fairly assess the cognitive status of people from different educational, cultural, and racial backgrounds (Manly, 2005; Sink et al., 2015; Carson et al., 2017). Given that the aim of this study was to investigate a diverse sample, we did not exclude participants who scored lower than 26 on the MOCA. Instead, we examined scores from both the neuropsychological assessment and experimental task to ensure that the participants could perform the tasks and did not suffer from cognitive impairment.

On the first lab visit, participants were given an accelerometer and an activity log. They were instructed to wear the ActiGraph wGT3X-BT accelerometer at all times (except for situations where water may damage the device such as when bathing or swimming).

**FIGURE 1:**
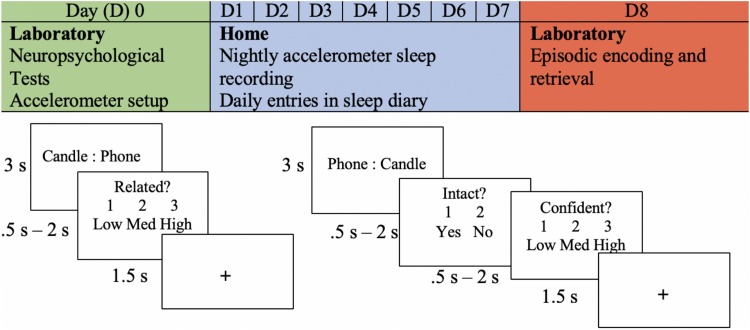
Experimental procedure. An example of the experimental procedure and the memory task is shown. The encoding task is shown in the bottom left, and retrieval is shown in the bottom right. The depicted trial is an example of an intact pairing.

They returned to the lab after a week of sleep measurement, and EEG was recorded as they performed an associative memory task (see Figure 1). Word stimuli for the memory task was chosen using two methods. In the first method, words were generated from the MRC Psycholinguistic Database: Machine Usable Dictionary, Version 2.00 (Wilson, 1988) according to general standards from previous studies (Dockree et al., 2015). All words ranged from 4 to 6 letters with a written frequency of 2 to 60. They had concrete and imaginability ratings of 300 to 700 (Frances and Kucera, 1982). We compiled 45 word pairs using this method and classified them into low, medium, and high similarity levels. For the second method, 207 word pairs were collected from a combination of databases that had empirically based similarity ratings (Finkelstein et al., 2002; Li et al., 2013; Szumlanski et al., 2013; Hill et al., 2015). The word pairs were divided into separate categories for low, medium, and high similarity. Similarity levels were counterbalanced across blocks of the encoding task.

Encoding was divided into four blocks. Each block consisted of 63 trials, and each trial began with a word pair that remained on the screen for 3 s. On the following screen, participants were prompted to determine the similarity between the word pair previously presented. This task was given to encourage deep encoding and to minimize the use of varying mnemonic strategies across participants. They were given the options of “1” for minimally related to “3” for highly related (indicated as low, medium, and high). Participants had a minimum of 0.5 s and a maximum of 2 s to make this decision. In other words, if a response was given before 0.5 s, the trial ended at 0.5 s; if a response was given after 0.5 s, the trial ended at that time. This method was chosen to allow participants who were slower enough time to respond, while also accommodating those who responded faster.

Participants began the intact/rearranged retrieval task shortly after encoding. Intact word pairs were presented with the same word that they were presented with at encoding, but the words in the first position were changed to the second position to avoid unitization of the word pairs (see Figure 1). Rearranged word pairs were presented with different words than paired with during the encoding task. No new words were presented at retrieval. One-third of the word pairs presented during encoding were rearranged, and two-thirds remained intact. This method was chosen to maximize the potential for data analysis, as only responses to intact word pairs were able to be compared to those during encoding. The task was divided into four blocks, each consisting of 63 trials. Each trial included an intact/rearranged decision and confidence judgement; participants were prompted to indicate their confidence in their response (low, medium, or high). The minimum time allotted for both responses was 0.5 s and the maximum was 2 s. There were three versions of the retrieval task that were counterbalanced across participants. Given that two-thirds of the pairs were intact and one-third of the pairs were rearranged, the third of the pairs that were rearranged were modified in each version. Thus, each pair was in the rearranged set in one of the versions.

After completion of the study participants were given a set of questionnaires to assess mood, caffeine intake, subjective sleep quality, and preferred time of day: the Pittsburg Sleep Quality Index (Buysse et al., 1989), Epworth Sleepiness Scale (Johns, 1991), the 21-item version of Depression, Anxiety, and Stress Scale (DASS-21; Lovibond and Lovibond, 1995), the Caffeine Intake Questionnaire (Landrum, 1992), and the Morningness Eveningness Questionnaire (Horne and Ostberg, 1976). Participants were later emailed an online questionnaire to assess experiences of race-related stress using the Index of Race-Related Stress-Brief (IRRS-B; Utsey and Ponterotto, 1996).

### EEG Acquisition

Electrophysiological signals were recorded from 32 Ag-AgCl electrodes using an ActiveTwo amplifier system (Biosemi, Amsterdam, Netherlands). Electrodes were positioned according to the extended 10–20 system (Nuwer et al., 1998). Electrodes were located at left/right hemisphere locations (FP1/FP2, AF3/AF4, F3/F4, F7/F8, FC1/FC2, FC5/FC6, C3/C4, T7/T8, CP1/CP2, CP5/CP6, P3/P4, P7/P8, PO3/PO4, O1/O2) and midline sites (Fz, Cz, Pz, Oz). Two electrodes were placed on the left and right mastoids for offline referencing. Vertical electrooculogram and horizontal electrooculogram were monitored by four additional electrodes placed above and below the right eye and on the outer canthus of each eye, respectively. The ActiveTwo system replaces traditional reference and ground electrodes with common mode sense and driven right leg electrodes, respectively. EEG was acquired with 24-bit resolution at a sampling rate of 512 Hz.

### EEG Analysis

Offline EEG preprocessing was performed using EEGLAB (Delorme and Makeig, 2004), ERPLAB (Lopez-Calderon and Luck, 2014), and FIELDTRIP (Oostenveld et al., 2011) toolboxes, and this process was based on previous research in our lab (see Strunk et al., 2017). Continuous data was resampled to 256 Hz, referenced to the average value of the left and right mastoids, and band-pass filtered between 0.5 and 125 Hz. The data was epoched to the stimulus presentation (i.e., appearance of word pair at 0 ms) from –1000 to 3500 ms. Then, independent components analysis was run for ocular artifact detection. The independent components analysis was run on the first 20 principle components of the head electrodes. Components that resembled ocular artifacts (e.g., eye blinks and horizontal eye movements) were manually removed. Then, an automatic rejection algorithm was applied in order to detect extreme voltage shifts across more than one electrode. Following this process, the data was visually inspected to assess the epochs selected for rejection, and any ocular, electrical, or muscle artifacts were manually removed. The frequency decomposition was performed using Morlet wavelets with linearly spaced frequencies between 2 and 40 Hz at 5 cycles. The frequencies of interests were defined as theta (4 to 7 Hz), alpha (8 to 12 Hz), and beta (16 to 26 Hz).

### Actigraphy Data

The sleep variables of interest were the means and variances (calculated as the squared standard deviation across each participant’s week of sleep) for TST, WASO, sleep fragmentation index (SFI), and number of awakenings. TST is the total minutes spent asleep, and WASO is the sum of minutes spent awake after initially falling asleep. The SFI is a measure of restlessness during sleep; the number of awakenings is the frequency of awakenings during sleep.

### Statistical Analysis

The statistical analyses were conducted using the Statistical Package of Social Sciences 24 (SPSS). In an effort to measure recollection-based memory, which is disproportionately affected by age and sleep (Drosopoulos et al., 2005; Koen and Yonelinas, 2014), memory performance was limited to *d’*, calculated as the standardized proportion of high confidence hits subtracted from the standardized proportion high confidence false alarms. High confidence memory decisions have been found to be sensitive to age-related differences (Shing et al., 2009; Fandakova et al., 2013).

We used analysis of variance (ANOVA) to determine age and racial group differences. Age and racial group were entered as independent variables, and the dependent variables, neuropsychological measures, sleep discontinuity (mean and variance), and *d’*, were separately assessed. Lastly, relationships between measures of sleep discontinuity, and dependent variables, *d’* and memory-related neural oscillations were examined. The PROCESS macro (Hayes, 2018) was used to determine if the relationships between sleep and memory were moderated by age group (young or old) or racial group (Black or White).

## Results

One older participant was not administered the MOCA because of having recently taken it. No participants were excluded for low MOCA scores, as all participants in the final sample were able to perform the experimental task, and the MOCA score did not reliably predict *d’* performance [*r*(47) = 0.034, *p* = 0.815]. Thus, we retained the final sample of 50 participants (20 young, 30 old).

### Principal Components Analysis

Because of the intercorrelations among the sleep variables, we used PCA with Varimax rotation to obtain discrete sleep components. We included all enrolled participants (81; 36 young, 45 older) to obtain reliable components. We ran separate PCAs for the means and variances of the sleep variables. PCA components were retained if the Eigenvalues exceeded 1. Each PCA resulted in two components for the sleep means and sleep variances. Components were extracted as regression variables to examine their relationships with memory and underlying neural oscillations. Since the components were largely representations of disturbed sleep and sleep time or general fragmentation, we will refer to the first component as sleep discontinuity and the second as sleep time (see Table 2).

**Table 2:** Sleep variable loadings for principal component analysis.

	Sleep Variable	Sleep Discontinuity	Sleep Time
**Mean**	Total Sleep Time	–0.078	**0.974**
	Wake After Sleep Onset	**0.919**	–0.203
	Sleep Fragmentation	**0.825**	–0.420
	Number of Awakenings	**0.909**	0.159
**Variance**	Total Sleep Time	**0.523**	0.504
	Wake After Sleep Onset	**0.736**	0.091
	Sleep Fragmentation	–0.022	**0.943**
	Number of Awakenings	**0.898**	–0.011


*The table displays the loadings for the sleep variables of interest. Bold text indicates the dominant variables.*

### Alpha Desynchronization Memory Success Effect

We assessed mean differences for the contrast between high confidence hits and misses using cluster-corrected permutations (Oostenveld et al., 2011). Memory success effects were not found in the theta or beta frequency band. There were significant mean differences across participants in the alpha frequency from 440 to 1360 ms [*t*(49) = –4.691, *p* = 0.001]. The effect extended over 28 electrodes. Considering the broad topography of this effect, we divided the electrodes into frontal (Fp1;Fp2;AF3;F3;FC1;FC2;FC5;FC6;Fz), central (T7;T8;C3;C4;CP1;CP2;CP5;CP6;Cz), and posterior (P7;P8;P3;P4;PO3;PO4;Pz;O1;O2;Oz) regions to allow for the assessment of distinct spatial regions related to episodic memory; these contrasts were all statistically significant (*p* ≤ 0.002). Each participant’s mean for these memory success effects (high confidence hit vs. miss) were used to examine correlations between *d’* and sleep discontinuity.

### Age and Racial Group Differences in Memory and Sleep Quality

An Age Group (Young, Old) × Race (Black, White) ANOVA revealed a main effect of Age for *d’* [*F*(1,46) = 5.96, *p* = 0.033, η^2^*partial* = 0.095]. There was no significant main effect of Race or interaction effect (*p* > 0.47). In the present sample, only the mean sleep time component demonstrated a significant main effect of Race (*p* = 0.02, η^2^*partial* = 0.115; see Table 1); however, all sleep components in the behavioral sample reached significant main effects of Race (*p* < 0.03), indicating worse sleep quality in Black participants. Thus, we examined each sleep component as an independent variable of interest for the following correlational analyses.

### Association Between Habitual Sleep Discontinuity and Memory Performance

**FIGURE 2:**
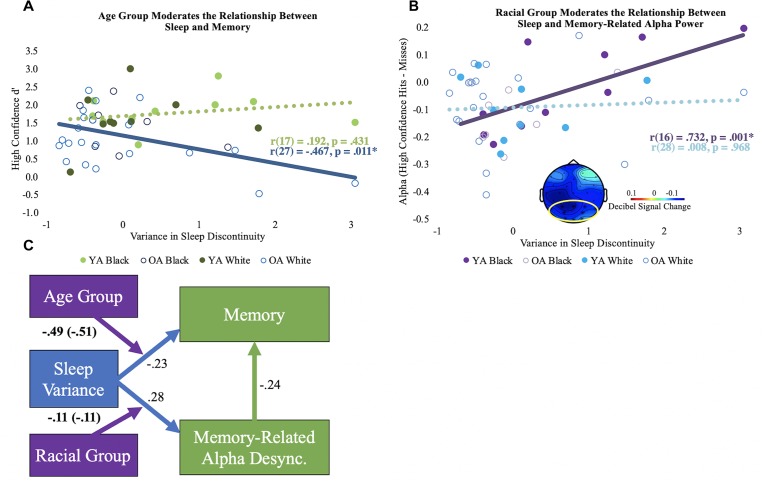
Moderation results. **(A)** Bivariate correlations (unadjusted) for the variance in sleep discontinuity and high confidence *d’* by age group. The significant correlation is shown using a solid line, and the non-significant correlation is shown using a dotted line. **(B)** Bivariate correlations (unadjusted) for the variance in sleep discontinuity and alpha desynchronization (high confidence hits minus misses) by racial group. The mean alpha desynchronization effect in the posterior electrode cluster (outlined in yellow; 440 to 1360 ms) is shown in bottom center of the plot. Each participant’s alpha power mean is shown. Significance is illustrated the same as in panel **(A)**. **(C)** Conceptual model of the moderation results. The direct paths for sleep discontinuity are shown in blue. The direct path for the correlation coefficient for memory-related alpha desynchronization and memory performance is depicted in green. Direct paths reflect the correlations across age and racial groups and are adjusted for chronological age. The moderator variables are shown in purple, and the age-adjusted interaction coefficients are displayed. Interaction coefficients that are not adjusted for age are in parentheses. Boldface indicates significant statistics. Desync = desynchronization.

After controlling for chronological age, sleep discontinuity variance correlated with *d’* in older adults [*r*(26) = –0.462, *p* = 0.013], but not young [*r*(16) = 0.168, *p* = 0.505]. Age group significantly moderated this relationship, even after controlling for years of age [Δ*R^2^* = 0.09, *F*(1, 43) = 5.30, *p* = 0.03; see Figure 2A]. No other moderation effects between the sleep components and *d’* reached statistical significance.

### Association Between Habitual Sleep Discontinuity and Alpha Desynchronization

After controlling for chronological age, sleep discontinuity variance significantly correlated with reduced memory-related alpha desynchronization for Black [*r*(15) = 0.685, *p* = 0.002, but not White *r*(27) = 0.007, *p* = 0.969] participants, particularly in posterior electrodes (see Figure 2B). Race significantly moderated this relationship after controlling for chronological age [Δ*R^2^* = 0.12, *F*(1, 43) = 6.75, *p* = 0.013]. No other moderation effects between alpha desynchronization and the sleep components were statistically significant.

### Age and Race-Related Differences in Moderation Effects

Taken together, the results demonstrate that intraindividual variance in habitual sleep discontinuity is linked to memory performance in older adults and memory-related alpha desynchronization in Black adults. A conceptual model of these findings is depicted in Figure 2C.

### Race-Related Stress

The racial relationships found above could be related to differences in socioeconomic status. Based on zip code census data, Black participants reported living in poorer areas than White participants [*t*(28) = –3.381, *p* = 0.001]. This analysis was limited to the older adult sample because the young adults were Georgia Tech students with similar zip codes. An Age Group (Young, Old) × Race (Black, White) ANOVA for the IRRS-B subscales demonstrated that Black participants, across age, reported more cultural, institutional, and individual experiences of race-related stress [*F*(1,16) = 10.09, *p* = 0.006, η^2^*partial* = 0.387; *F*(1,16) = 5.23, *p* = 0.035, η^2^*partial* = 0.249; *F*(1,15) = 8.33, *p* = 0.011, η^2^*partial* = 0.357]. Furthermore, variance in SFI was positively related to both cultural and individual race-related stress after controlling for chronological age [*r*(15) = 0.522; *r*(15) = 0.498, *p* < 0.05]. There were no racial group differences in the general stress variable measured by the DASS [*F*(1,46) = 1.51, *p* = 0.226, η^2^*partial* = 0.032].

## Discussion

The results of the present study reveal interesting interactions among variance in sleep discontinuity, associative memory, aging, and race. Consistent with previous research (Hicken et al., 2013; Slopen and Williams, 2014; Cunningham et al., 2016; Turner et al., 2016), we found numerical differences across measures of sleep discontinuity, illustrating that Black adults maintained poorer sleep quality than White adults. Differing from prior studies, we investigated the effects of the variance in sleep quality, or the changes in night-to-night sleep stability, on memory. We found that greater variance in sleep quality predicted worse memory performance in older adults, but not young. Sleep variance was also associated with reductions in the memory success effect of alpha desynchronization in Black adults, but not White adults, across age. In addition, neuropsychological measures of cognitive performance (e.g., MOCA) were not reliable predictors of high confidence memory performance, but sleep discontinuity was a reliable predictor. This result supports the notion that excluding participants using such measures may unnecessarily reduce racial/ethnic diversity in cognitively normal samples (Sink et al., 2015; Carson et al., 2017). These novel findings suggest that maintaining good sleep quality is especially important for memory in older adults and Black adults.

Older adults may be more sensitive to sleep quality because of age-related neuropathology such that age-related neural changes and poor sleep act as an additive effect to reduce memory performance (for a review see, Scullin, 2017). Moreover, race may moderate the relationship between sleep discontinuity and memory-related alpha desynchronization not inherently because of race, but because of race-related psychosocial factors. For example, we found that older, Black participants reported living in poorer areas, and Black adults across age reported experiencing more race-related stress. Moreover, stress levels have been found to affect confidence in memory, which could be related to a greater reliance on familiarity than recollection (Corbett et al., 2017). Greater sleep discontinuity in Black adults could be explained by interactions between stressful experiences of discrimination and health problems (e.g., greater body weight, diabetes, and heart disease) that commonly affect minority groups (Karlsen and Nazroo, 2002; Williams et al., 2015). Consistent with our findings, meta-analysis results show that more variance in sleep quality is often found in minority groups and young adults, and it is often associated with poor physical and psychological health (Bei et al., 2016).

Nonetheless, research for night-to-night variance in sleep quality is particularly scarce, and the studies that have examined sleep variance are often limited to subjective, self-report measures (Bei et al., 2016). It should be noted that there are often discrepancies between self-report sleep measures and objective sleep measures, especially for night-time awakenings (King et al., 2017). Furthermore, relationships between natural sleep patterns and the neural underpinnings of memory are largely unknown. Most of the sleep and memory research is focused on sleep manipulations instead of habitual sleep quality, and there are no studies (to our knowledge) that investigate interactions between objective sleep quality, age, and race in relation to memory performance. Given that both minorities and older adults tend to sleep more poorly than other populations, it is surprising that these relationships have not been investigated. Future research should include race as a variable of interest in their studies, considering that the present study found that Black adults had poorer sleep and a relationship between sleep and memory-related neural activity.

This pilot study is limited by its small sample size. It is also limited by its cross-sectional nature; thus, we cannot determine a causal relationship among the examined factors.

Although sleep difficulties are often found in older adults and minorities (Ohayon et al., 2004; Bei et al., 2016), the memory-related neural consequences of these effects are poorly understood. The results of this study highlight the importance of objectively investigating habitual sleep quality in larger, more diverse samples.

## Contribution to the Field Statement

At least one in every three adults regularly obtains insufficient amounts of sleep, and those who do not maintain good sleep quality are disproportionately older adults, racial/ethnic minorities, or both older and of minority status. However, very little is known about the neurocognitive consequences of poor, objectively measured habitual sleep quality, specifically its effect on episodic memory. No prior study has investigated this relationship with both age and race as variables of interest. The current study found that older adults may be more sensitive to sleep discontinuity, as their memory performance was worse when there was greater variance in measures of sleep disruption. Furthermore, Black adults slept more poorly than White adults, and they demonstrated a relationship with memory-related neural oscillations and sleep discontinuity such that greater night-to-night variance in sleep discontinuity weakened memory-related neural activity. Thus, the results from the current study suggest that poor habitual sleep quality may have differential effects on memory depending upon age and racial group. These findings highlight the necessity of further investigating the relationship between habitual sleep quality and episodic memory in diverse samples.

## Data Availability

The datasets generated for this study are available on request to the corresponding author.

## Ethics Statement

This study was carried out in accordance with the recommendations of the Central Institutional Review Board (IRB) with written informed consent from all subjects. All subjects gave written informed consent in accordance with the IRB. The protocol was approved by the Georgia Tech IRB.

## Author Contributions

EH contributed to the conceptual design of the study and collected and analyzed the data. AD guided the conceptualization of the study and the interpretation of the results. EH and AD wrote the manuscript.

## Conflict of Interest Statement

The authors declare that the research was conducted in the absence of any commercial or financial relationships that could be construed as a potential conflict of interest.
